# Association of Radiographic Signs in Determining the Proximity of Mandibular Third Molar Roots to the Mandibular Canal and Postoperative Occurrence of Neurosensory Disorders: A Cohort Study

**DOI:** 10.7759/cureus.51085

**Published:** 2023-12-25

**Authors:** Vijayendranath Nayak, Sameer Kumar, Silpa Madhuri, Karthik Kannaiyan, Melwin Mathew, Htoo htoo kyaw Soe, Preethy mary Donald, Manasa Anand Meundi, Saptarshi Bhowal

**Affiliations:** 1 Oral Medicine and Oral Radiology, Manipal University College Malaysia, Melaka, MYS; 2 Oral and Maxillofacial Pathology, Manipal University College Malaysia, Melaka, MYS; 3 Prosthodontics, Manipal University College Malaysia, Melaka, MYS; 4 Periodontics, Manipal University College Malaysia, Melaka, MYS; 5 Community Medicine, Manipal University College Malaysia, Melaka, MYS; 6 General Dentistry, Manipal University College Malaysia, Melaka, MYS

**Keywords:** mandibular canal, inferior alveolar nerve, inferior alveolar canal, mandibular third molar, neurosensory disorders

## Abstract

Background: The routine oral and maxillofacial procedure involving the surgical removal of impacted mandibular third molars comes with inherent risks to nearby anatomical structures. Proximity of mandibular third molar roots to the inferior alveolar nerve (IAN) poses a significant risk for injury, prompting the need for reliable assessment methods. Radiographic indicators, particularly those observed on intraoral periapical radiographs (IOPARs), offer a dependable means to evaluate proximity.

Objectives: This study seeks to examine the closeness between the mandibular canal and the roots of mandibular third molars using IOPARs and to assess the incidence of postoperative neurosensory disorders.

Methods: A cohort of 100 subjects aged 18 to 25, presenting for partially erupted/ impacted mandibular third molar removal, underwent IOPAR examinations. Data analysis employed IBM SPSS Statistics for Windows, Version 12 (Released 2004; IBM Corp., Armonk, New York, United States), calculating frequencies, percentages, means, standard deviations, and ranges. Radiographic signs of proximity were evaluated, and a standardized surgical procedure was performed under local anesthesia. Postoperative neurosensory disorders were assessed using various methods.

Results: Of the evaluated subjects, darkening of the root (52%) was the most prevalent radiographic sign, followed by interruption of the white line of the canal (20%). The prevalence of radiographic signs varied, with none of the patients experiencing narrowing of the root. Postsurgical paraesthesia assessment revealed no nerve sensitivity alterations in any patient.

Conclusion: Preoperative radiographic examination is imperative for determining the relationship between mandibular third molar roots and the inferior alveolar canal, aiding in preventing IAN damage during extraction. Contrary to radiographic signs, there was no observed association between impacted mandibular third molar radiographic signs and the occurrence of postoperative neurosensory disorders.

## Introduction

The routine oral and maxillofacial procedure involving the surgical removal of impacted mandibular third molars comes with inherent risks to nearby anatomical structures [1]. The most common risk factor for injury is the juxtaposition of the roots of the mandibular third molar to the inferior alveolar nerve (IAN) [2]. There are specific signs of proximity observed on an intraoral periapical radiograph (IOPAR) which is a dependable method for estimating the proximity of the impacted mandibular third molar and the mandibular canal (MC) [3]. Rood and Shehab formatted seven radiographic indicators of a close relationship between the mandibular third molar roots and the inferior alveolar canal (IAC) [4]. In this study, IOPARs were employed to evaluate the relationship between the MC and the roots of the mandibular third molar, as well as to assess the occurrence of neurosensory disorders postoperatively.

## Materials and methods

This study included 100 participants aged between 18 and 25 years from the Southern part of India, who were attending the Department of Oral Medicine and Radiology (A. J. Institute of Medical Sciences and Research Centre, Mangalore, India), for an IOPAR referred by the Department of Oral and Maxillofacial Surgery for partially erupted/ impacted mandibular third molar removal.

Inclusion criteria

The study included individuals meeting the criteria of having a partially erupted/impacted mandibular third molar with the presence of the mandibular second molar and a good-quality radiograph.

Exclusion criteria

The study excluded radiographs where the IAC was not identified. Individuals with root displacement resulting from pathological conditions (such as cysts or tumors) and with systemic illnesses and pregnant participants were excluded.

Data analysis

The data was analyzed using IBM SPSS Statistics for Windows, Version 12 (Released 2004; IBM Corp., Armonk, New York, United States). For categorical variables such as gender and prevalence of radiographic signs, frequency and percentage were calculated, while mean, standard deviation, and range were calculated for quantitative variables such as age. A bar chart was used to display the prevalence of radiographic signs of proximity in the patients with impacted third molars using IOPARs.

Preoperative clinical and radiographic examination

**Figure 1:**
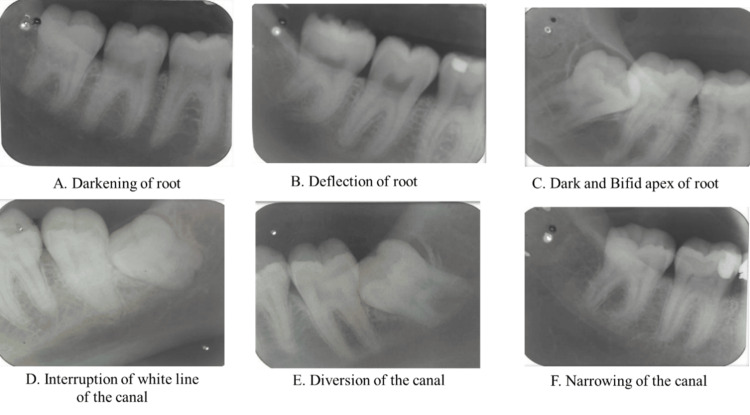
Intraoral periapical radiographs showing radiographic signs of proximity

Patients with partially erupted/impacted mandibular third molar referred by the Department of Oral Maxillofacial Surgery were subjected to IOPARs after preliminary clinical examination and were taken into the study. Informed written consent was taken from the patient and the procedure was explained to the patient. The subjects were instructed to don a lead apron and sit in a conventional dental chair, ensuring that the sagittal plane was perpendicular and the occlusal plane parallel to the floor. Utilizing the bisecting angle technique, a single intraoral periapical Kodak E-speed film was captured. Patients were briefed on the procedure and instructed to maintain stability during the exposure. The film was placed in snap a ray holder with all the exposure parameters set (70kvp, 7mA, and 0.7 sec), and the exposure was made. All preoperative radiographs were examined for the presence of the seven radiological signs: darkening of the root (DR), deflection of the root, narrowing of the root (NR), the dark and bifid apex of the root (DBR), interruption of the white line of the canal (IWL), diversion of the canal (DC), and narrowing of the canal (NC) (Figure 1).

Only 40 subjects showed radiographic signs of proximity and were considered for the study. The surgical procedure adhered to standardized protocols and was conducted within the Department of Oral and Maxillofacial Surgery under local anesthesia, employing suitable instruments and stringent infection control measures. Subsequent to surgery, patients were monitored for the development of postoperative neurosensory disorders. Assessments were conducted on the first day postoperatively and during a follow-up appointment 7 to 10 days later, utilizing tools such as cotton wool, a blunt probe, and a pinprick to evaluate sensation.

## Results

In the present study, the maximum age of patients was 25, and the minimum was 19 years. The mean age of the patients was 22.5 years (SD 1.57) and 57.5% were female (Table 1).

**Table 1:** Demographic characteristics of patients.

Variable	N (%)
Age (years)	
Mean (SD)	22.5 (1.57)
Minimum - Maximum	19 - 25
Gender	
Male	17 (42.5)
Female	23 (57.5)

IOPARs were evaluated for the radiographic signs of proximity, in which DR was seen in maximum followed by IWL, NC, DC, DFR, and DBR. NR was absent in the evaluated subjects (Table 2).

**Table 2:** Percentage of radiographic signs of proximity.

Radiographic Signs of Proximity	Number	Percentage
Darkening of root	21	52%
Deflection of root	3	7%
Narrowing of the root	0	0
Dark and bifid apex of the root	1	3%
Interruption of the white line of the canal	8	20%
Diversion of the canal	3	8%
Narrowing of the canal	4	10%
Total	40	100%

**Figure 2:**
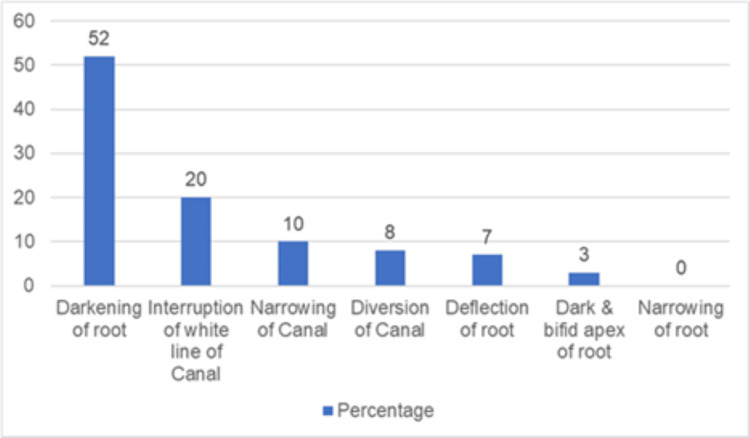
Prevalence of radiographic signs.

The prevalence of radiographic signs of proximity in the patients with impacted third molars was evaluated using IOPARs. The highest prevalence was DR (n=21, 52%) followed by IWL (n=8, 20%), NC (n=4, 10%), DC (n=3, 8%), deflection of the root (n=7, 7%), and DBR (n=1, 3%). None of the patients had a NR (0%) (Figure 2).

Upon analyzing the outcomes of the techniques employed to assess postsurgical paresthesia, it was noted that none of the patients exhibited any alterations in nerve sensitivity. These findings can be juxtaposed with the information provided in Table 3 of the published data.

**Table 3:** Prospective studies about the incidence <1 % of neurosensory injury to IAN after surgical removal of mandibular third molars. IAN: Inferior alveolar nerve

Author	Country	Injury to IAN (%)
Goldberg et al. [5]	USA	0.6
Middlehurst et al. [6]	UK	0
Obiechina [7]	Nigeria	0.82
Absi and Shepherd [8]	UK	0.91
Berge and Boe [9]	Norway	0.49
Bell [10]	UK	0
Benediktsdottir et al. [11]	Iceland	0.52
Costa et al. [12]	Brazil	0
Present study	India	0

## Discussion

Surgical extraction of mandibular third molars is the most common minor surgical procedure carried out in dental practice [5]. Few complications may be encountered during the postoperative period. One of them is postsurgical sensory impairment [6]. The iatrogenic origin of neurosensory dysfunction is a distressing sequel to the surgical removal of impacted mandibular third molars, which is frequently overlooked [13]. The risk of injuring the inferior alveolar neurovascular bundle is increased when the anatomic relation between the root tip and mandibular canal is not exactly determined. Hence determining the position of the mandibular canal in relation to the root tip of impacted mandibular third molars is of utmost importance [14]. Thus, accurate knowledge of the position of the impacted teeth may contribute to the feasibility of the surgical approach as well as determining the prognosis of impaction [15].

The preoperative radiographic evaluation has been considered as having potential capacity to predict possible injuries to the IAN during a surgical procedure [12]. Many authors have suggested radiographic signs, prior to surgical removal of mandibular third molars that are in close relationship with IAN [3,10,12].

In the current study, DR was noticed in 52% of the cases, followed by IWL seen in 20 % of the cases. NC was seen in 10 % of the cases followed by DC with 8 % prevalence, DR with 7 % prevalence, and DBR was seen only in 3 % of the cases. A study conducted by Sinha et al. (2015) showed DR in 48 % of the cases which had a high prevalence followed by IWL in 32 % of the cases which correlated with the result of our study [16].

Sedaghatfar et al. identified a statistically noteworthy correlation between four radiographic signs: DR, NR, IWL, and DC, with the risk of intraoperative IAC exposure [17].

According to Howe and Poyton's findings, a genuine correlation with the IAN exists in 93% of third molars displaying radiographic root darkening [18]. Additionally, Bell demonstrated that the risk of IAN exposure during the removal of third molars increased to 11% when the white line of the canal was interrupted and to 52% when there was radiographic darkening of the roots [10]. However, in this study, none of the cases showed any exposure to IAN.

Apart from the proximity of roots of mandibular third molars to the IAN, surgeons' experience, patients' age, tissue manipulation, and postsurgical edema can also be reasons for IAN injury [17].

Alternative treatment modalities like coronectomy or intentional partial odontotomy of third molars have been shown to reduce the risk of an IAN deficit when the third molar root is in close proximity to the IAN [19,20]. Using advanced imaging techniques, preoperative prediction of neurovascular bundle exposure is extremely useful for warning patients of the potential risk of postoperative dysesthesia and obtaining informed consent [21].

## Conclusions

A prerequisite for preoperative radiographic assessment is essential to ascertain the correlation between the roots of the mandibular third molar and the IAC, facilitating the prevention of IAN damage during the extraction of the mandibular third molar.

Based on these current findings, we conclude that there is no association between radiographic signs of impacted mandibular third molars with mandibular canal proximity and post-operative neurosensory disorders occurrence. Utilizing CBCT imaging can serve as an effective means to assess the three-dimensional anatomical proximity between the mandibular third molar and the IAC, thereby preventing potential damage to the IAN during the extraction of the mandibular third molar in patients.
